# Terpolymer Donor with Inside Alkyl Substituents on Thiophene *π*‐Bridges toward Thiazolothiazole A_2_‐Unit Enables 18.21% Efficiency of Polymer Solar Cells

**DOI:** 10.1002/advs.202203513

**Published:** 2022-10-31

**Authors:** Liuyang Zhou, Lei Meng, Jinyuan Zhang, Shucheng Qin, Jianqi Zhang, Xiaojun Li, Jing Li, Zhixiang Wei, Yongfang Li

**Affiliations:** ^1^ Beijing National Laboratory for Molecular Sciences CAS Key Laboratory of Organic Solids Institute of Chemistry Chinese Academy of Sciences Beijing 100190 China; ^2^ School of Chemical Science University of Chinese Academy of Sciences Beijing 100049 China; ^3^ CAS Key Laboratory of Nanosystem and Hierarchical Fabrication National Center for Nanoscience and Technology Beijing 100190 China; ^4^ Key Laboratory of Photochemical Conversion and Optoelectronic Materials Technical Institute of Physics and Chemistry Chinese Academy of Sciences Beijing 100190 China; ^5^ Laboratory of Advanced Optoelectronic Materials College of Chemistry Chemical Engineering and Materials Science Soochow University Suzhou Jiangsu 215123 China

**Keywords:** D–A copolymer donors, PM6‐based terpolymers, polymer solar cells, side chain position on thiophene *π*‐bridges, ternary random copolymers

## Abstract

PM6 is a widely used D–A copolymer donor in the polymer solar cells (PSCs). Incorporating second electron‐withdrawing (A_2_) units into PM6 backbone by ternary D–A_1_–D–A_2_ random copolymerization strategy is an effective approach to further improve its photovoltaic performance. Here, the authors synthesize the PM6‐based terpolymers by introducing thiazolothiazole as the A_2_ units connecting with thiophene π‐bridges attaching alkyl substituent towards the A_2_ unit (PMT‐CT) or towards D‐unit (PMT‐FT), and study the effect of the alkyl substituent position on the photovoltaic performance of them. Two terpolymers PMT‐FT‐10 and PMT‐CT‐10 are obtained by incorporating 10% A_2_ units in the terpolymers. The film of PMT‐CT‐10 shows slightly up‐shifted highest occupied molecular orbital (HOMO) energy levels while better co‐planar structure than that of PMT‐FT‐10. Meanwhile, the PMT‐CT‐10:Y6 blend film exhibits better molecular packing properties, more proper phase separation and more balanced hole and electron mobilities, which are beneficial to more efficient exciton dissociation, efficient charge transport and weaker bimolecular recombination. Consequently, the PMT‐CT‐10 based PSCs obtain the highest power conversion efficiency of 18.21%. The results indicate that side chain position on the thiophene π‐bridges influence the device performance of the terpolymer donors, and PMT‐CT‐10 is a high efficiency polymer donor for the PSCs.

## Graphical abstract

Two PM6‐based D–A_1_–D–A_2_ type terpolymers PMT‐FT‐10 and PMT‐CT‐10 are synthesized by introducing 10% thiazolothiazole as the second electron‐accepting (A_2_) units connecting with thiophene *π*‐bridges attaching alkyl substituent toward the A_2_ unit (PMT‐CT‐10) or toward D‐unit (PMT‐FT‐10). The polymer solar cells with PMT‐CT‐10 as donor and Y6 as acceptor show the highest power conversion efficiency of 18.21%.

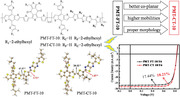

## 1 Introduction

Polymer solar cells (PSCs) with a p‐type conjugated polymer as electron donor and an n‐type organic semiconductor as electron acceptor, have become a promising technology for converting sunlight to electricity due to their unique properties of simple device structure, low‐cost solution processing, light‐weight, and flexibility.^[^ ^1^, ^2^, ^3^, ^4^, ^5^, ^6^, ^7^, ^8^, ^9^, ^10^, ^11^ ^]^ In the last decade, the PSCs have made tremendous progress, mainly benefited from the development of non‐fullerene small molecular acceptors (SMAs).^[^ ^12^, ^13^, ^14^, ^15^, ^16^, ^17^, ^18^ ^]^ Especially, the emergence of Y6,^[^ ^18^ ^]^ an A–DA′D–A‐structured SMA explored by Zou et al., brings PSCs into a new era. The power conversion efficiency (PCE) of the PSCs has achieved over 18% recently, with Y6 or its derivatives as acceptor and wide bandgap p‐type conjugated polymer as donor.^[^ ^19^, ^20^, ^21^, ^22^, ^23^, ^24^ ^]^ Among the high performance polymer donors, PM6^[^ ^25^ ^]^ is a representative and widely used polymer donor for the PSCs with Y6 or its derivatives as acceptors. PM6 is a D–A copolymer with bi(alkyl‐fluoro‐thienyl)‐benzodithiophene (BDT‐F) as D‐unit and 1,3‐bis(thiophen‐2‐yl)‐5,7‐bis(2‐ethylhexyl)benzo[1,2‐c:4,5‐c0]dithiophene‐4,8‐dione (BDD) as A‐unit.

In order to further improve the photovoltaic performance of PM6, ternary random copolymerization strategy has been employed recently,^[^ ^26^, ^27^, ^28^, ^29^, ^30^ ^]^ which introduces a third component of either D_2_^[^ ^31^, ^32^, ^33^ ^]^ or A_2_^[^ ^34^, ^35^, ^36^, ^37^, ^38^ ^]^ into PM6 binary D–A backbone to form D_1_–A–D_2_–A or D–A_1_–D–A_2_ type terpolymers, respectively. By careful controlling the proportion of the third component, the optimized terpolymers could effectively promote PCEs of the PSCs by synergistically improving light absorption, tuning energy levels, and controlling morphology and crystallinity of the polymer donors. Recently, Cao et al.^[^ ^39^ ^]^ reported a D–A_1_–D–A_2_ type ternary random copolymer PFBCNT20 by introducing 3,4‐dicyanothiophene (DCT) as A_2_ unit into PM6 backbone with 20% DCT. And a higher PCE of 16.3% was obtained for the PSCs with PFBCNT20 as donor. In 2020, Yan et al.^[^ ^40^ ^]^ synthesized a series of PM6‐based D–A_1_–D–A_2_ type terpolymers donor polymers with difluorobenzo[c][1,2,5]thiadiazole, *p*‐difluorobenzene, tetrafluorobenzene, or thiazolo[5,4‐d]thiazole as A2 unit, and the PCEs of the PSCs with the terpolymers as donor reached 16–17.1%. In the same year, Zhang et al.^[^ ^41^ ^]^ incorporated thiazolo[5,4‐d]thiazole (TTz) as the A_2_ unit into the PM6 backbone, and synthesized terpolymer PM1 with 20% A_2_ proportion. PM1 possesses near‐perfect co‐planar structure, downshifted the highest occupied molecular orbital (HOMO) energy level, enhanced the crystallinity, and improved morphology in comparison with PM6, and the PM1‐based PSC achieved high PCE of 17.6%. All of the abovementioned A_2_ units connecting with the alkyl substituted thiophene as *π*‐bridges where the alkyl chains are far from A_2_ unit (toward D‐unit). While for the A_2_ unit of TTz without substituents, there could be some steric hindrance for the substituents of thiophene *π*‐bridges toward D‐units, and the alkyl substituent toward TTz A_2_ unit should be beneficial for more perfect co‐planar structure and more efficient charge transportation of the terpolymers.

**Figure 1:**
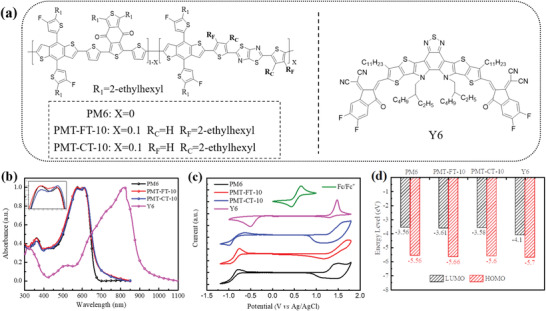
a) Chemical structures of polymer donors and Y6 acceptor. b) Normalized absorption spectra of the neat polymer donors and Y6 films. c) Cyclic voltammograms of polymer donors and Y6. d) Schematic energy level diagram of the polymer donors and Y6 acceptor.

Based on this consideration, we synthesized a new D–A_1_–D–A_2_ type terpolymer PMT‐CT‐10 (see **Figure** **1**a) with 10% TTz A_2_ unit connected with the thiophene *π*‐bridges attaching alkyl substituent toward the TTz A_2_ units, and another D–A_1_–D–A_2_ type terpolymer PMT‐FT‐10 (see Figure 1a) with 10% TTz A_2_ unit connected with the thiophene *π*‐bridges attaching alkyl substituent toward the D units for comparison. PMT‐CT‐10 exhibits better co‐planar molecular structure and higher absorption coefficient, while slightly upshifted the HOMO energy level than PMT‐FT‐10. Eventually, the PMT‐CT‐10‐based PSC with Y6 as acceptor achieves excellent PCE of 18.21%, with an open‐circuit voltage (*V*_oc_) of 0.832 V, an outstanding short‐circuit current density (*J*_sc_) of 28.12 mA cm^−2^, and a higher fill factor (FF) of 77.9%, which is one of the highest PCE among the terpolymer‐based PSCs. While, the PMT‐FT‐10‐based device yields a moderate PCE of 17.44%, with a *V*_oc_ of 0.838 V, a *J*_sc_ of 27.11 mA cm^−2^, and a FF of 76.8%.

## 2 Results and Discussion

The target terpolymers were synthesized via the Stille coupling reaction, from the two commonly used monomers for PM6, including the electron‐rich4,8‐bis(5‐(2‐ethylhexyl)‐4‐fluorothiophen‐2‐yl)benzo[1,2‐*b*:4,5‐*b*′]‐dithiophene (BDT‐F) D‐unit and electron‐deficient1,3‐bis(thiophen‐2‐yl)‐5,7‐bis(2‐ethylhexyl)benzo‐[1,2‐*c*:4,5‐*c*′]dithiophene‐4,8‐dione (BDD) A1‐unit, and the TTz A_2_ unit connecting with thiophene *π*‐bridges attaching alkyl substituent close to A_2_ units (TTz‐CT‐Br) or far from A_2_ unit (toward D units)(TTz‐FT‐Br). The detailed synthetic routes and synthetic processes of the monomers TTz‐FT‐Br and TTz‐CT‐Br and the two polymer donors PMT‐FT‐10 and PMT‐CT‐10 are shown in Scheme S1a,b, Supporting Information. And the synthetic routes of the PMT‐CT‐based terpolymers with 20%, 30%, and 100% proportion of A_2_ units are displayed in Scheme S1b, Supporting Information. At room temperature, the resulting polymer donors show good solubility in common organic solvents, such as chloroform and chlorobenzene. The number‐average molecular weights (*M*_n_) of polymer donors were measured by high‐temperature gel permeation chromatography with 1,3,5‐trichlorobenzene as the eluent. The *M*_n_ of PM6, PMT‐FT‐10, and PMT‐CT‐10 were obtained to be 36.80, 35.40, and 37.84 KDa, with corresponding polydispersity index of 2.13, 1.76, and 2.49, respectively (shown in **Table** **1** and Figure S1, Supporting Information). Meanwhile, all the three polymer donors demonstrate good thermal stability with 5% weight loss at temperature about 415 °C by thermogravimetric analysis (Figure S2a, Supporting Information).

**Table 1:** Molecular weights, optical properties, and electronic energy levels of polymer donors

Polymer donor	*M*_n_ [kDa]	PDI	$\lambda_{\max}^{\mathrm{film}}$ [nm]	$\lambda_{\mathrm{onset}}^{\mathrm{film}}$ [nm]	$E_{g}^{\mathrm{opt}.}$ [eV]^a)^	*E*_HOMO_ [eV]^b)^	*E*_LUMO_ [eV]^b)^
PM6	36.80	2.13	575, 612	665	1.86	−5.56	−3.56
PMT‐FT‐10	35.40	1.76	576, 612	680	1.82	−5.66	−3.61
PMT‐CT‐10	37.84	2.49	578, 613	685	1.81	−5.60	−3.58

^a)^ Optical band‐gap was calculated from $E_{g}^{\mathrm{opt}}$ = 1240/*λ*_onset_;

^b)^ Estimated from the onset oxidation/reduction potentials on the CV curves.

Figure 1b shows the normalized UV–vis absorption spectra of the three polymer donors and Y6 films, and Figure S2b, Supporting Information, displays the normalized UV–vis absorption spectra of the polymer donors in diluted chloroform solution. And the corresponding optical properties data are summarized in Table 1. In the solution state, the three polymer donors display almost identical absorption profiles with similar maximum absorption peaks at 615 nm and a shoulder peak at about 578 nm, while the shoulder peak of the two terpolymers is enhanced than PM6, indicating the stronger interchain interactions for the two terpolymers. In the film state, the three polymers exhibit similar absorption peaks with maximum absorption peaks approximately at 576 and 612 nm, respectively, which indicates that there are strong aggregations of the three polymers. The shoulder peak at ≈576 nm is further enhanced in the thin films than that in solutions (see the inset of Figure 1b), indicating stronger interchain interaction in the two terpolymer films. It is worth mentioning that the absorption peak of PMT‐CT‐10 film is slightly broadened and its absorption coefficient at the peak wavelength of 612 nm is enhanced than PM6 and PMT‐FT‐10 (see Figure S2c, Supporting Information), indicating the enhanced interchain interaction and stronger light harvesting ability of PMT‐CT‐10. PMT‐FT‐10 and PMT‐CT‐10 display absorption edges at 680 and 685 nm corresponding to the optical bandgap of 1.82 and 1.81 eV, respectively. Furthermore, the normalized absorption profiles of blend films of the three polymer donors and Y6 acceptor exhibit similar absorption profiles (see Figure S2d, Supporting Information). While compared to PM6 and PMT‐FT‐10‐based blends, the PMT‐CT‐10‐based blend film possesses relatively higher absorption coefficient (Figure S2e, Supporting Information), which could be beneficial to improve photon harvesting efficiency. Based on above discussion, we could draw the conclusion that the terpolymer PMT‐CT‐10 possesses stronger absorption and interchain interaction, indicating its better planar molecular structure.

Electrochemical cyclic voltammetry (CV) was employed to estimate the electronic energy levels of the polymer donors, and the cyclic voltammograms (CV curves) are shown in Figure 1c. The HOMO and the lowest unoccupied molecular orbital (LUMO) energy levels were calculated according to the equations of *E*_HOMO/LUMO_ = −e (*φ*_ox/red_ + 4.80 − *φ*_Fc/Fc_^+^) (eV), where the *φ*_ox/red_ represent the onset oxidation (*φ*_ox_) and onset reduction (*φ*_red_) potentials obtained from CV curves with the unit of V versus Ag/AgCl, and the *φ*_Fc/Fc_^+^ was measured to be 0.44 V versus Ag/AgCl. Finally, the equations could be expressed as *E*_HOMO/LUMO_ = *−e* (*φ*_ox/red_ + 4.36) (eV), and the *E*_HOMO_/*E*_LUMO_ values of PM6, PMT‐FT‐10, and PMT‐CT‐10 were calculated to be −5.56/−3.56, −5.66/−3.61, and −5.60/−3.58 eV, respectively, as shown in Table 1 and Figure 1d. The energy levels of the two terpolymers are downshifted a little compared with PM6, and PMT‐CT‐10 shows slightly higher (0.06 eV) HOMO energy level than PMT‐FT‐10.

**Figure 2:**
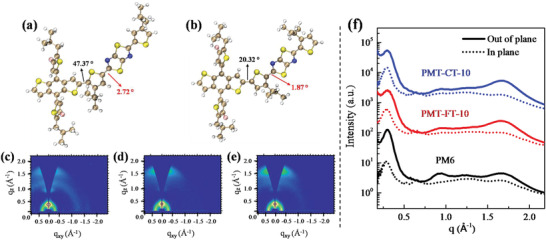
DFT calculated torsion angles of the optimized molecular geometry of a) PMT‐FT‐10 and b) PMT‐CT‐10. 2D‐GIWAXS images of c) PM6, d) PMT‐FT‐10, e) PMT‐CT‐10, and f) corresponding 1D line‐cuts of neat films in the in‐plane (IP) and out‐of‐plane (OOP) direction.

In order to investigate the effect of the alkyl substituents positions on the thiophene *π*‐bridges on the molecular planarity of the terpolymers, the optimal geometries of shortened molecule of D‐T‐A_2_‐T (where T represents thiophene *π*‐bridges) for PMT‐FT‐10 and PMT‐CT‐10 with simplified side chains were calculated by density functional theory (DFT) (Figure S3a–d, Supporting Information). As shown in **Figure** **2**a,b, the dihedral angles for PMT‐CT‐10 between the alkyl substituted thiophene *π*‐bridges and the BDT‐F D‐unit, and TTz A_2_ unit are 20.32° and 1.87°, respectively, which are significantly smaller than those of 49.37° and 2.72° for PMT‐FT‐10. The result indicates that there is big steric hindrance for the alkyl substituents on the thiophene *π*‐bridges far from the A_2_ units (close to the D‐units) in PMT‐FT‐10.

Grazing incident wide‐angle X‐ray scattering (GIWAXS) measurement was conducted on the neat polymer donors to investigate their molecular packing properties, and all the original polymer films were prepared under identical conditions. The 2D GIWAXS patterns and corresponding 1D intensity profiles are shown in Figure 2c–f, and the fitting results are summarized in Table S5, Supporting Information. Pronounced *π*–*π* stacking (010) diffraction peaks are observed in the out‐of‐plane (OOP) direction for PMT‐FT‐10 and PMT‐CT‐10 films, indicating that they have more preferential orientations. The (010) diffraction peaks are located at 1.62 and 1.64 Å^−1^, corresponding to the *π*–*π* stacking distance of 3.88 and 3.83 Å for PMT‐FT‐10 and PMT‐CT‐10, respectively. The corresponding crystal coherence length (CCL) of PMT‐FT‐10 and PMT‐CT‐10 is calculated to be 10.89 and 11.24 Å, respectively (see Table S5, Supporting Information). The closer *π*–*π* stacking and longer CCL for PMT‐CT‐10 should be beneficial for the charge transportation in the PSC devices. In addition, in comparison with the *d*‐spacing of 3.98Å of PM6, the two terpolymers of PMT‐FT‐10 and PMT‐CT‐10 possess smaller *d*‐spacing values of their *π*–*π* stacking, indicating that the ternary random copolymerization is an effective strategy for improving the *π*–*π* stacking aggregation of the polymer donors.

**Figure 3:**
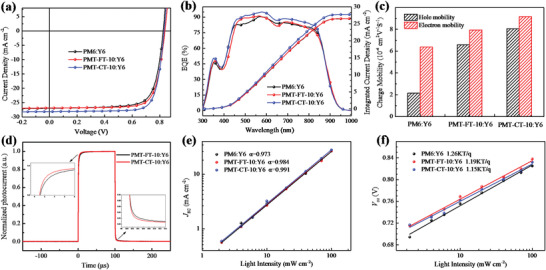
a) *J–V* curves of the optimized PSCs under illumination of AM1.5G, 100 mW cm^−2^. b) EQE curves and corresponding integrated current densities of the PSCs. c) The determined charge carrier mobilities of the blend films. d) Normalized transient photocurrent (TPC) of the terpolymers‐based devices in response to a 100 µs white‐light (LED) pulse for light intensity of 100 mA cm^−2^. e) *J*_sc_ and f) *V*_oc_ dependence on the light intensity of the corresponding PSCs.

To investigate the effect of the alkyl substitution position of the thiophene *π*‐bridges on the photovoltaic performance of the terpolymers, PSCs were fabricated with conventional device architecture of ITO/PEDOT:PSS/Polymer donors:Y6/PNDIT‐F_3_N^[^ ^42^ ^]^/Ag. First, the device fabrication conditions of the PMT‐CT‐10‐based devices were optimized by tuning the D/A weight ratio (w/w), treatment conditions of additive, and thermal annealing.^[^ ^43^ ^]^ Meanwhile, the photovoltaic properties of the other terpolymers PMT‐CT‐20, PMT‐CT‐30, and PMT‐CT‐100 with 20%, 30%, and 100% proportion of TTz‐CT units, respectively, were studied by using Y6 as acceptor under the optimized device fabrication conditions. Table S1, Supporting Information, summarized the photovoltaic performance data of the PSCs. It can be seen that the optimized device fabrication conditions are the D:A weight ratio of 1:1.2, 0.5% CN additive treatment, and thermal annealing at 110 °C for 10 min, and PMT‐CT‐10 shows the best photovoltaic performance among the terpolymers containing different proportion of TTz A_2_ units. **Figure** **3**a shows the current density–voltage (*J*–*V*) curves of the optimized devices, and the corresponding photovoltaic parameters are listed in **Table** **2**. The PMT‐CT‐10‐based PSC shows the highest PCE of 18.21% with a high *J*_sc_ of 28.12 mA cm^−2^ and a higher FF of 77.9% while a slightly lower *V*_oc_ of 0.832 V due to the slightly upshifted HOMO energy levels of the PMT‐CT‐10 donor, in comparison with that of the PMT‐FT‐10‐based device. Meanwhile, PMT‐FT‐10‐based device also reached a higher PCE of 17.44% with *V*_oc_ of 0.838 V, *J*_sc_ of 27.11 mA cm^−2^ and FF of 76.8%, compared with that of PM6 (PCE of 16.72%, *V*_oc_ of 0.825 V, *J*_sc_ of 26.95 mA cm^−2^, FF of 75.3%).

**Table 2:** Photovoltaic performances parameters of the optimized PSCs with thermal annealing treatment at 110 °C for 10 min, under the illumination of AM1.5G, 100 mW cm^−2^

Donor:acceptor^a)^	*V*_oc_ [V]^b)^	*J*_sc_ [mA cm^−2^]^b)^	FF [%]^b)^	PCE [%]^b)^	*J*_sc_ [EQE] [mA cm^−2^]^c)^
PM6:Y6	0.825 (0.823 ± 0.002)	26.95 (26.72 ± 0.19)	75.3 (74.6 ± 0.6)	16.72 (16.58 ± 0.14)	26.53
PMT‐FT‐10:Y6	0.838 (0.835 ± 0.004)	27.11 (26.84 ± 0.23)	76.8 (76.3 ± 0.7)	17.44 (17.25 ± 0.16)	26.56
PMT‐CT‐10:Y6	0.832 (0.830 ± 0.002)	28.12 (27.96 ± 0.15)	77.9 (77.4 ± 0.4)	18.21 (18.11 ± 0.11)	27.78

^a)^ All devices were fabricated with D/A weight ratio of 1:1.2 and with 0.5% CN additive;

^b)^ The average values with standard deviation in parentheses were obtained from more than 15 devices;

^c)^ The integrated *J*_sc_ from the EQE curves.

In addition, photovoltaic performance of the PSC based on PMT‐CT‐10 with different molecular weight was measured and the corresponding photovoltaic performance data are summarized in Table S2, Supporting Information. All the devices show decent PCE of exceeding 17.3%, which indicates that PMT‐CT‐10 possesses good reproducibility. Meanwhile, the PMT‐CT‐10‐based PSCs with different active layer thicknesses are fabricated, and the corresponding *J–V* curves are displayed in Figure S4, Supporting Information, and the photovoltaic parameters are summarized in Table S3, Supporting Information. The resulting PSCs exhibit the insensitivity to the thickness of active layer, and the PSC with 300 nm active layer still shows relatively good PCE of 15.46%.

Figure 3b displays the external quantum efficiency (EQE) spectra of the optimized devices, and the integrated current densities from the EQE spectra for the corresponding devices are also listed in Table 2. All the optimized PSCs show high and similar photoresponse profiles in the wavelength range from 300 to 940 nm, and the PMT‐CT‐10‐based device demonstrates higher EQE responses in the whole wavelength range. The *J*_sc_ values calculated from the integration of EQE profiles are 26.53, 26.56, and 27.78 mA cm^−2^ for the devices based on PM6, PMT‐FT‐10, and PMT‐CT‐10 respectively, which agrees well with those obtained from their *J–V* curves.

To deeply probe the effect of alkyl substitution position of the thiophene *π*‐bridges on the charge transport properties of the terpolymers, the electron mobility (*µ*_e_) and hole mobility (*µ*_h_) of the polymer donor films and their blend films with Y6 acceptor were measured by space‐charge‐limited‐current method, with the device structures of ITO/ZnO/blend films/PNDIT‐F_3_N/Ag for electron mobility measurement and ITO/PEDOT:PSS/neat films or blend films/MoO_3_/Ag for hole mobility measurement. The results are shown in Figure S5a–c, Supporting Information, and corresponding charge carrier mobilities are summarized in Figure 3c and Figure S5d and Table S4, Supporting Information. After incorporating A_2_ units, the *µ*_h_ of two terpolymers are improved compared with PM6, and the PMT‐CT‐10 film possesses the highest *µ*_h_ of 7.74 × 10^−4^ cm^2^ V^−1^ s^−1^ due probably to its better molecular packing property. After blending with Y6 acceptor, the PMT‐CT‐10‐based blend film also displays the highest *µ*_h_ of 8.06 × 10^−4^ cm^2^ V^−1^ s^−1^ and *µ*_e_ of 9.16 × 10^−4^ cm^2^ V^−1^ s^−1^ with more balanced electron and hole mobilities (*µ*_e_/*µ*_h_) of 1.14. The PMT‐FT‐10‐based blend film with Y6 acceptor shows slightly lower *µ*_h_ (6.58 × 10^−4^ cm^2^ V^−1^ s^−1^) and *µ*_e_ (7.94 × 10^−4^ cm^2^ V^−1^ s^−1^) with a *µ*_e_/*µ*_h_ value of 1.21, which are also higher than those of the PM6:Y6 blend (*µ*_h_ = 2.16 × 10^−4^ cm^2^ V^−1^ s^−1^, *µ*_e_ = 6.37 × 10^−4^ cm^2^ V^−1^ s^−1^ with an unbalanced *µ*_e_/*µ*_h_ of 2.93). The results indicate that PMT‐CT‐10‐based blend film with Y6 acceptor possesses the highest and the balanced hole and electron mobilities, which should be beneficial to the higher FF of the PMT‐CT‐10‐based PSCs.

Furthermore, transient photocurrent measurements were carried out to investigate the charge extraction and recombination behavior of the PSCs. As shown in Figure 3d, the PMT‐CT‐10‐based device shows fastest rise and fall response to the light pulse, implying efficient charge extraction and reduced charge recombination. The results are fully consistent with the more efficient and balanced charge transport properties of the PMT‐CT‐10‐based device. Meanwhile, the dependence of carrier lifetime (*τ*) on carrier density (*n*) was plotted and fitted in Figure S6a, Supporting Information; the PMT‐CT‐10‐based device possesses longer charge carrier lifetime and higher carrier density, indicating lower carrier recombination rate in PMT‐CT‐10‐based device. Furthermore, the bimolecular recombination rate constants (*k*_rec_) are calculated from the carrier lifetime and carrier density with the formula of *k*_rec_ = 1/([*λ*+]) *nτ*), meanwhile, the *τ* obey a power law relationship with *n* of *τ* = *τ*_0_ *n* ^−*λ*^, where the exponential factor *λ* is extracted from Figure S6a, Supporting Information. As shown in Figure S6b, Supporting Information, the PMT‐CT‐10‐based device shows lower *k*_rec_ than the other two devices at various light intensities, which indicates the faster charge extraction and the reduced bimolecular recombination in the PMT‐CT‐10‐based PSCs. The results are consistent with the relatively higher FF of the PMT‐CT‐10‐based PSCs.

To explore the charge transport and charge recombination behavior in the active layers, the dependence of *J*_sc_ on light intensity (*P*_light_) was further studied. Generally, *J*_sc_ and *P*_light_ follow the relationship of *J*_sc_ ∝ *P*_light_^*α*^,^[^ ^44^ ^]^ where the exponential factor *α* will approach to 1 if the bimolecular recombination is negligible. As shown in the plots of log (*J*_sc_) versus log (*P*_light_) in Figure 3e, the slope *α* values were calculated to be 0.973, 0.984, and 0.991, corresponding to the devices based on PM6:Y6, PMT‐FT‐10:Y6, and PMT‐CT‐10:Y6, respectively. The *α* value of 0.991 for the PMT‐CT‐10:Y6‐based PSCs is closest to 1, indicating the weaker bimolecular recombination, which agrees well with its better photovoltaic performances. In addition, the dependence of *V*_oc_ on *P*_light_ was studied to further investigate the charge recombination mechanism. In general, the slope of *V*_oc_ versus ln (*P*_light_) should be close to *kT*/*q* (where *k* is the Boltzmann constant, *T* is the Kelvin temperature, and *q* is the elementary charge) when the bimolecular recombination dominates. As shown in Figure 3f, the slopes were calculated to be 1.26 *kT*/*q*, 1.19 *kT*/*q*, and 1.15 *kT*/*q*, for the PSCs based on PM6:Y6, PMT‐FT‐10:Y6, and PMT‐CT‐10:Y6, respectively. The slope of the PMT‐CT‐10:Y6‐based PSCs is closer to *kT*/*q* among the three devices, suggesting the bimolecular recombination is the predominant process. The results indicate that the PSCs based on PMT‐FT‐10 could better suppress the bimolecular recombination and more efficient charge transportation, which are beneficial to obtain the higher FF and better device performance for the PMT‐CT‐10‐based PSCs.

The photoluminescence (PL) quenching experiment was also carried out to further investigate the exciton dissociation in the blend films based on the terpolymers:Y6. As shown in Figure S7, Supporting Information, all neat terpolymer films displayed strong PL emissions in the range of 600–≈800 nm when excited at 590 nm, but the PL emissions of the terpolymer donors were almost completely quenched in the blend films with Y6 acceptor. The PMT‐CT‐10:Y6 blend film shows higher quenching efficiency of 98.25% than that (97.62%) of the PMT‐FT‐10‐based film, which further indicates the more efficient exciton dissociation in the PMT‐CT‐10:Y6 blend film.

**Figure 4:**
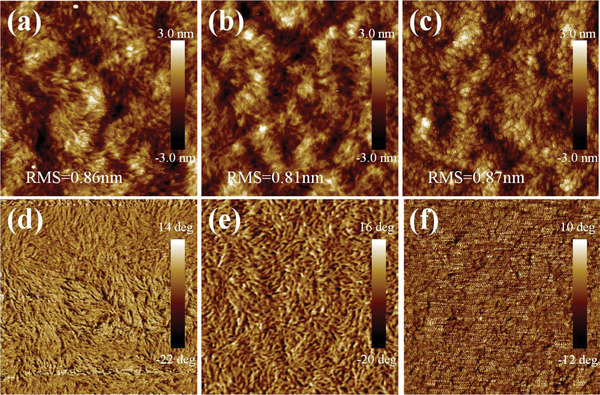
a–c) AFM height images (1 × 1 µm^2^) and d–f) phase images (1 × 1 µm^2^) of the blend films based on: a,d) PM6:Y6; b,e) PMT‐FT‐10:Y6; c,f) PMT‐CT‐10:Y6.

To further understand the effect of alkyl substitution position on the morphological characteristics of the terpolymers, the atomic force microscopy (AFM) and transmission electron microscopy (TEM) were carried out to investigate the surface and bulk morphology of the blend films based on PM6:Y6, PMT‐FT‐10:Y6, and PMT‐CT‐10:Y6. **Figure** **4**a–f shows the AFM height images and corresponding phase images with the size of 1 × 1 µm^2^ for the blend films, and Figure S8a–c, Supporting Information, shows the height images of the blend films with 5 × 5 µm^2^ size. As shown in Figure 4c, the PMT‐CT‐10‐based blend film exhibits fiber network morphology with a slightly larger root‐mean‐square roughness value of 0.87 nm than the other two blend films. Furthermore, the phase images of PM6 and PMT‐FT‐10‐based blend films show rod‐like texture morphology. Whereas the PMT‐CT‐10‐based phase image shows continuous interpenetrating network morphology with more proper phase separation, which benefits the efficient exciton dissociation and charge transport. In addition, the TEM images (Figure S8d–f, Supporting Information) display similar trend, suitable phase separation was observed in the terpolymer‐based blend films by bright and dark domains, which further indicates that the ternary random copolymerization strategy could effectively improve phase separation and result in increasing FF for the corresponding PSCs.

**Figure 5:**
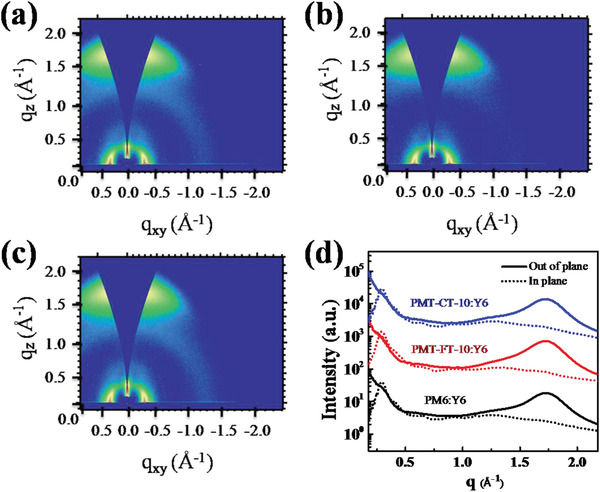
2D‐GIWAXS images of the blend films based on a) PM6:Y6, b) PMT‐FT‐10:Y6, and c) PMT‐CT‐10:Y6. d) Corresponding 1D line‐cuts in the in‐plane (IP) and out‐of‐plane (OOP) direction.

The GIWAXS measurements of blend films were carried out to further investigate the molecular packing behavior. The 2D GIWAXS images and corresponding 1D line‐cuts are shown in **Figure** **5**a–d and Figure S9a,b, Supporting Information, and the fitting results are summarized in Table S6, Supporting Information. All blend films exhibit pronounced *π*–*π* stacking (010) diffraction peaks in the OOP direction, indicating preferential face‐on orientations in the vertical direction of substrate. The PMT‐FT‐10 and PMT‐CT‐10‐based blend films show the similar (010) diffraction peaks with slightly smaller *π*–*π* stacking distance (*d*) in the OOP direction than the PM6‐based blends, indicating relatively tighter molecular packing in the terpolymer‐based blend films. Furthermore, PMT‐FT‐10 and PMT‐CT‐10‐based blend films possess larger CCLs of 17.78 and 18.24 Å than the PM6‐based blends (17.62 Å) in OOP direction (Table S6, Supporting Information), which indicates the enhanced aggregation on the face‐on orientation. The results show that the ternary random copolymerization strategy plays an important role in tuning the molecular packing and aggregation of the blend films. And PMT‐CT‐10 with the alkyl substituent toward TTz A_2_ unit possesses better molecular packing and face on aggregation, which are beneficial for the higher FF and better photovoltaic performance of the PMT‐CT‐10‐based PSCs.

**Figure 6:**
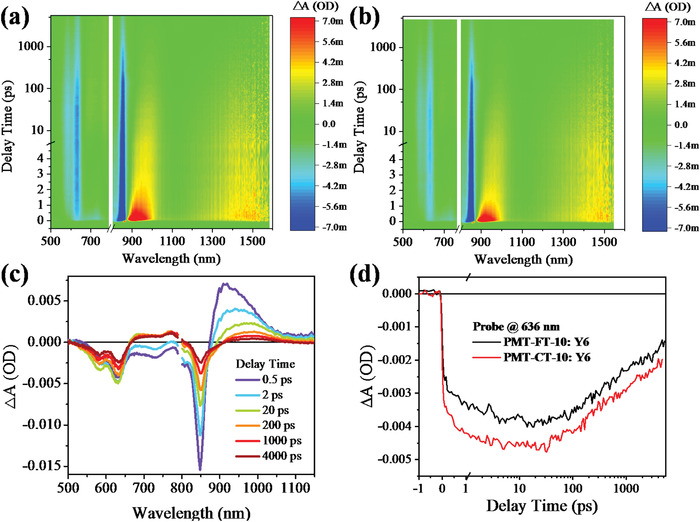
2D transient absorption spectra of a) PMT‐CT‐10:Y6 and b) PMT‐FT‐10:Y6 blend films with excitation at 830 nm. c) Transient absorption spectra of the PMT‐CT‐10:Y6 blend film at selected time delays. d) Kinetic traces of the terpolymer donors GSB probing at 636 nm for the two blend films.

To further investigate the charge transfer (CT) dynamics in the active layer, we performed femtosecond transient absorption spectroscopy on the PMT‐CT‐10:Y6 and PMT‐FT‐10:Y6 blend films to probe the photo‐induced hole transfer processes.^[^ ^45^ ^]^ First, we measured the transient absorption spectra of the PMT‐CT‐10:Y6 blend by selectively photoexciting the Y6 acceptor in the blend film with pump wavelength at 830 nm. As shown in **Figure** **6**a,c, a broad ground state bleach (GSB) signal with a peak at 850 nm was observed immediately after the excitation, resulted from the Y6 excitons. Another strong GSB peak centered at 636 nm appears almost simultaneously, which is ascribed to the GSB of polymer donor since it matches the absorption of the PMT‐CT‐10 film. The formation of this GSB is the result of ultrafast hole transfer from Y6 exciton to PMT‐CT‐10 at the D/A interface in the blend. The intensity of donor GSB signal continued to increase to its maximum in ≈30 ps, and then it started to decrease slowly. The features of CT state decay to non‐zero over 4 ns time‐window of our instrument, indicating a slow charge recombination of the CT state in the active layer. The long‐lived CT state provided sufficient time for charge migration and collection at the electrodes, which is crucial for high photocurrent in the OSC devices. Then the transient absorption spectra of PMT‐FT‐10:Y6 was also measured and analyzed under the same condition (Figure 6b). Since the two terpolymers both display steady‐state absorption with a peak at 636 nm, the kinetic traces at this wavelength were extracted from their transient spectra and compared in Figure 6d. It is clear that PMT‐CT‐10 blends exhibits a more intense GSB signal than the PMT‐FT‐10 blends, suggesting a high yield in the hole transfer process, and therefore resulting in a better photocurrent generation in the PMT‐CT‐10 blends. These findings agree well with the excellent performances of the PMT‐CT‐10‐based devices.

In conclusion, two PM6‐based D–A_1_–D–A_2_‐type terpolymers were designed and synthesized by introducing 10% TTz as the A_2_ units connecting with thiophene *π*‐bridges attaching alkyl substituent toward the A_2_ unit (PMT‐CT‐10) or toward D‐unit (PMT‐FT‐10), for the application as donor in PSCs. Among the two terpolymers, PMT‐CT‐10 film exhibits less steric hindrance, more planar molecular structure, stronger light harvesting ability, and better molecular packing properties. And the blend films of PMT‐CT‐10 with acceptor Y6 show suitable D/A interpenetrating network, enhanced *π*–*π* stacking and face‐on orientation, higher and balanced hole and electron mobilities, and higher excitons dissociation and less charge recombination efficiencies, which leads to the enhanced *J*_sc_ and higher FF in the PMT‐CT‐10:Y6‐based PSCs. Finally, the PMT‐CT‐10:Y6‐based PSCs achieves the highest PCE of 18.21%, which is one of the highest efficiencies reported among the terpolymers‐based PSCs. The results indicate that the alkyl substitution position plays an important role in tuning the aggregation and interchain interaction of the polymer donors, and PMT‐CT‐10 is a high performance polymer donor for the Y6‐based PSCs.

## 3 Experimental Section

The detailed synthesis process and characterization of polymer donors, the device fabrication, and characterization can be found in the Supporting Information.

### Statistical Analysis

The pre‐processing of data included normalization, transformation, image merging, and integration. Data were reported as the mean ± SD. Sample size for each statistical analysis is described in the corresponding table. In the experimental process, the control variable method was generally employed to reduce the experimental error. Statistical analysis was performed using software such as Origin 2018 and Nanoscope Analysis.

## Conflict of Interest

The authors declare no conflict of interest.

## Supporting information

Supporting Information

## Data Availability

The data that support the findings of this study are available in the supplementary material of this article.
