# Supplementary material for: Terpolymer Donor with Inside Alkyl Substituents on Thiophene π‐Bridges toward Thiazolothiazole A2‐Unit Enables 18.21% Efficiency of Polymer Solar Cells
Source: Adv Sci (Weinh). 2022 Oct 31;9(34):2203513. doi: 10.1002/advs.202203513 (PMC9731682; doi:10.1002/advs.202203513)
Supplement: Supplementary file 1 — Supporting Information [file ADVS-9-2203513-s001.pdf]

ADVANCED SCIENCE
Open Access

Supporting Information

for *Adv. Sci.*, DOI 10.1002/advs.202203513

Terpolymer Donor with Inside Alkyl Substituents on Thiophene π-Bridges toward Thiazolothiazole A_2-Unit Enables 18.21% Efficiency of Polymer Solar Cells

Liuyang Zhou, Lei Meng, Jinyuan Zhang, Shucheng Qin, Jianqi Zhang, Xiaojun Li, Jing Li, Zhixiang Wei and Yongfang Li**

Supporting Information

Terpolymer Donor with Inside Alkyl Substituents on Thiophene π-Bridges towards Thiazolothiazole A_2-unit Enables 18.21% Efficiency of polymer solar cells

Liuyang Zhou[a,b], Lei Meng[a,b], Jinyuan Zhang[a], Shucheng Qin[a,b], Jianqi Zhang[d], Xiaojun Li[a,b], Jing Li[e], Zhixiang Wei[d], Yongfang Li[a,b,c*]*

[a] *Beijing National Laboratory for Molecular Sciences, CAS Key Laboratory of Organic Solids, Institute of Chemistry, Chinese Academy of Sciences, Beijing, 100190, China*

[b] *School of Chemical Science, University of Chinese Academy of Sciences, Beijing, 100049,China*

[c] *Laboratory of Advanced Optoelectronic Materials, College of Chemistry, Chemical Engineering and Materials Science, Soochow University, Suzhou, Jiangsu, 215123, China*

[d] *CAS Key Laboratory of Nanosystem and Hierarchical Fabrication, National Center for Nanoscience and Technology, Beijing, 100190, China*

[e] *Key Laboratory of Photochemical Conversion and Optoelectronic Materials, Technical Institute of Physics and Chemistry, Chinese Academy of Sciences, Beijing 100190, China*

***Corresponding authors:**

menglei@iccas.ac.cn (L.Meng), liyf@iccas.ac.cn (Y. Li)

Experimental Section

Materials: Compounds BDT-F and BDD (**Scheme S1b**) were purchased from Solarmer Materials Inc.; TTz-FT and TTz-CT were commercially available from Nanjing ZhiYan Technology Co. Toluene was dried over Na/benzophenone and freshly distilled prior to use. The other chemicals, solvents and materials used in this work were all commercially available and used without further purification.

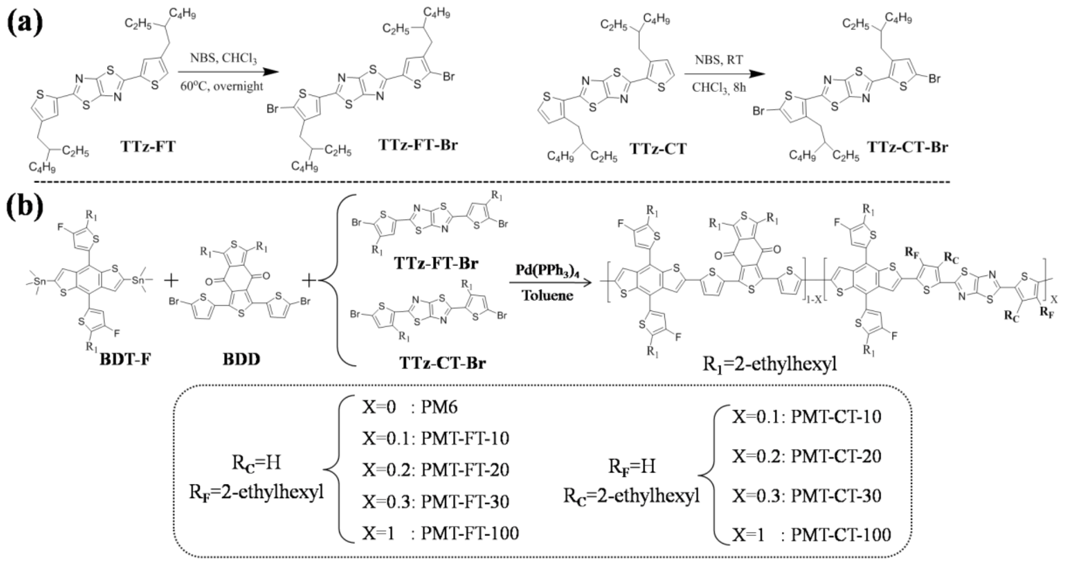

SchemeS1. Synthetic routes of a) TTz-FT-Br, TTz-CT-Br and b) polymer donors with incorporating A_2 units of different proportions.

Detailed synthesis process:

2,5-bis(5-bromo-4-(2-ethylhexyl)thiophen-2-yl)thiazolo[5,4-d]thiazole (TTz-FT-Br):

TTz-FT (0.94 mmol, 0.5 g) was dissolved in 50 mL $CHCl_3$ under Ar atmosphere with 100 ml round-bottom flask, and stirred at 60 ℃ for 20 min. Then, N-Bromosuccinimide (NBS) (2.82 mmol, 0.51 g) was dissolved in DMF and added in one portion. After the solution was stirred at 60℃ overnight, the mixture solution was poured into water (200 mL) to quench the reaction. The organic phase was extracted with CH_2Cl_2 and washed by water and brine for three times and after drying over $MgSO_4$. The combined organic phase was obtained and the solvent was removed and purified with silica column chromatography using petroleum ether and

dichloromethane (*v/v*=2:1) as eluent, then further purification was carried out by recrystallization from methyl alcohol/dichloromethane. Finally, the pure compound was obtained as lamellar yellow solid. (0.48 g, yield: 74.3%)

^{1}H NMR (400 MHz, CDCl3) δ 7.22 (s, 2H), 2.52 (d, J = 7.2 Hz, 4H), 1.70–1.63 (m, 2H), 1.37–1.27 (m, 16H), 0.95–0.88 (m, 12H).

^{13}C NMR (101 MHz, CDCl3) δ 160.78, 148.75, 141.72, 135.65, 126.70, 112.98, 38.97, 32.90, 31.47, 27.76, 24.71, 22.02, 13.07, 9.81.

MS (MALDI-TOF): m/z 687.02 (M^+).

2,5-bis(5-bromo-3-(2-ethylhexyl)thiophen-2-yl)thiazolo[5,4-d]thiazole (TTz-CT-Br):

TTz-CT (0.94 mmol, 0.5 g) was dissolved in 50 mL $CHCl_3$ with 100 ml single-port round-bottom flask, and stirred at atmosphere for 20 min. Then, N-Bromosuccinimide (NBS) (2.07 mmol, 0.37 g) was dissolved in DMF and added in one portion. After the solution was stirred at room temperature for 8 h, the mixture solution was poured into water (200 mL) to quench the reaction. The organic phase was extracted with CH_2Cl_2 and washed by water and brine for three times and after drying over $MgSO_4$. The combined organic phase was obtained and the solvent was removed and purified with silica column chromatography using petroleum ether and dichloromethane (*v/v*=3:1) as eluent, then further purification was carried out by recrystallization from methyl alcohol/dichloromethane. Finally, the pure compound was obtained as needle-like yellow solid. (0.56 g, yield: 86.7%)

^{1}H NMR (400 MHz, $CDCl_3$) δ 6.91 (s, 2H), 2.84 (d, *J* = 7.3 Hz, 4H), 1.76–1.68 (m, 2H), 1.41–1.25 (m, 16H), 0.92–0.86 (m, 12H).

^{13}C NMR (101 MHz, CDCl3) δ 160.34, 149.98, 142.77, 134.10, 133.89, 115.26, 39.89, 34.56, 32.56, 28.66, 25.71, 23.04, 14.10, 10.67.

MS (MALDI-TOF): m/z 687.02 (M^+).

Poly[(2,6-(4,8-bis(5-(2-ethylhexyl-3-fluoro)thiophen-2-yl)-benzo[1,2b:4,5-b']dithiophene))-alt-(5,5-(1',3'-di-2-thienyl-5',7'-bis(2-ethylhexyl)benzo[1',2'-c:4',5'c']dithiophene-4,8-dione)] (PM6)

BDT-F monomer (0.1 mmol, 94.05 mg) and BDD monomer (0.1 mmol, 76.67 mg) were dissolved in dry toluene (5 mL) and added into a two-necked flask. The solution was flushed with Ar for 20 min and then the $Pd(PPh_3)_4$ (6 mg) was added into the mixtures, the solution was flushed with Ar again for another10 min. The oil bath was gradually heated to 110℃ and the reactant was stirred for 24h at this temperature. After the reaction mixture was cooled to room temperature, the mixture was poured into methanol (150 ml) slowly, allowing the crude polymer to precipitate. After filtration, the crude product was subjected to Soxhlet extraction with methanol, hexane and chloroform in sequence. The chloroform fraction was evaporated and further dried in a vacuum over night to get the final product as a black solid.

Synthesis of terpolymers

BDT-F monomer (0.1 mmol, 94.05 mg), corresponding BDD and A2 unit monomers with corresponding proportions were added into a two-necked flask and dissolved by dry toluene (5 mL). The solution was flushed with Ar for 20 min and then the $Pd(PPh_3)_4$ (6 mg) was added into the mixtures, the solution was flushed with Ar again for another10 min. Meanwhile, the oil bath was gradually heated to 110℃ and the reactant was stirred for 24h at this temperature. The further purification was carried with the same procedure as described above. Finally, we further dried in a vacuum over night before use, and got the final product as a black solid.

Yield: PMT-FT-10: 69.8%; PMT-CT-10: 72.3%.

Measurements and instruments

^{1}H NMR and ^{13}C NMR spectra were recorded on Bruker Avance 400 MHz NMR spectrometer at room temperature (Shown in **Figure S10-S13**). The UV-vis absorption spectra were measured on a UH4150 Spectrophotometer (Direct Light Detector). Cyclic voltammogram (CV) measurements were conducted on a Zahner IM6e electrochemical workstation using sample film coated Platinum disk electrode as the working electrode, Pt wire as the counter electrode, and Ag/AgCl as the

reference electrode in a 0.1 M tetrabutylammonium hexafluorophosphate (Bu_4NPF_6) acetonitrile solution. Fc/Fc^+ redox couple was used as the inner reference for the calculation of the electronic energy levels of the polymer donor and acceptor materials.

Transient absorption spectroscopy (TAS)

Femtosecond transient absorption spectrometer was composed of a regenerative-amplified Ti: sapphire laser system (Coherent) and Helios pump-probe system (Ultrafast Systems). The regenerative-amplified Ti: sapphire laser system (Legend Elite-1K-HE, center wavelength of 800 nm, pulse duration of 25 fs, pulse energy of 4 mJ, repetition rate of 1 kHz) was seeded with a mode-locked Ti: sapphire laser system (Vitara) and pumped with a Nd: YLF laser (Evolution 30). The output 800 nm fundamental of the amplifier was split into two beam pulses. The main part of the fundamental beam went through the optical parametric amplifiers (TOPAS-C), whose output light was set as the pump light with wavelength of 830 nm and chopped by a mechanical chopper operating at frequency of 500 Hz. A small part of the fundamental beam was introduced into the TA spectrometer in order to generate the probe light. After passing through a motorized optical delay line, the fundamental beam was focused on a sapphire crystal or YAG crystal, which was used to generate the white-light continuum (WLC) probe pulses with wavelength of 430 to 820 nm or 800 to 1600 nm, respectively. The optical path difference between the pump light and the probe light, which is controlled by the motorized optical delay-line, was used to monitor the transient states at different pump-probe delay. A reference beam was split from the WLC in order to correct the pulse-to-pulse fluctuation of the WLC. The pump was spatially and temporally overlapped with the probe beam on the sample. Excitation energy of the pump pulse was set to 2 $\mu J/cm^2$ to avoid singlet-singlet annihilation. The film samples for TA measurements were prepared by spin coating the corresponding materials on thin quartz plates. The film samples were thermally annealed the same way as the actual device.

Device fabrication and characterization

Polymer solar cells (PSCs) were fabricated with the conventional device structure of ITO/PEDOT:PSS (30 nm)/Polymer donors:Y6 (125 nm)/PNDIT-F_3N/Ag (100 nm). Firstly, the ITO-coated glass substrates were washed by sequential ultrasonication in water/detergent, secondary water, acetone, and isopropanol for 16 min in each solvent; then, the washed substrates were further operated with UV-O_3 cleaner for 25 min, after that the PEDOT:PSS (Baytron P VP AI 4083 from H.C. Starck) aqueous solution was spin-coated onto the ITO substrate at a spin rate of 7000 rpm for 30 s and then annealing in air at 150°C for 20 min. The blends of Polymer donors:Y6 processed by $CHCl_3$ (with the addition of 0.5% CN (*v/v*)) at a concentration of 18 mg mL^{-1} was prepared by stirring at room temperature for 2 hour. Then, the polymer blends were spin-coated on the top of PEDOT:PSS layer at the speed of 4500 rpm to achieve the optimized film thickness. The dried films were thermally annealed at 110°C for 10 min inside nitrogen glove box, and cooling after thermal annealing. After that, a cathode buffer layer of PNDIT-F_3N with a concentration of 0.5 mg ml^{-1} was spin-coated on the surface of active layer with the thickness of 10 nm. Finally, a silver electrode (Ag, 100 nm) was vacuum-deposited at 1.0×10^{-6} Pa.

Photovoltaic measurements: The current density-voltage (*J*-*V*) characteristics were measured in glove box on a computer-controlled Keithley 2450 Source-Measure Unit with the active layer area of 0.06 mm^2. OrielSol3A Class AAA Solar Simulator (model, Newport 94023A) with a 450 W xenon lamp and an air mass (AM) 1.5 filter was used as the light source. The scanned voltage is from -1.2 V to 1.0 V. The external quantum efficiency (EQE) was measured by Solar Cell Spectral Response Measurement System QE-R3-011 (Enli Technology Co., Ltd., Taiwan).

Mobility measurements: The hole and electron mobilities were measured using the space charge limited current (SCLC) method. Hole-only and electron-only devices were fabricated with ITO/PEDOT:PSS/active layer/MoO_3/Ag and ITO/ZnO/active layer/PNDIT-F_3N/Ag structures, respectively. The SCLC mobilities were calculated

by MOTT-Gurney equation (1):

$$J = \frac{9\varepsilon_r\varepsilon_0\mu V_{eff}^2}{8L^3} \quad (1)$$

where J is the current density, ε_r is the relative permittivity of the active layer material, ε_0 is the permittivity of free space, μ is the charge (hole or electron) mobility at zero filed, L is the thickness of the active layer, V_{eff} is the effective voltage (V_{eff}=V_0-V_{bias}).

GIWAXS characterization

Grazing-incidence wide-angle X-ray scattering (GIWAXS) measurements were carried out on a Xeuss 2.0 SAXS/WAXS system (Xenocs SA, France) with X-ray wavelength of 1.5418 Å. The samples were prepared on Si substrates using blend solutions identical to those used in devices and irradiated at a fixed angle of 0.3°.

AFM and TEM measurements

The morphologies of the blend films were investigated by atomic force microscopy (AFM, Agilent Technologies, 5500 AFM/SPM System, USA) in contacting mode with a 1 μm or 5 μm scanner. Transmission electron microscope (TEM) measurements were performed in a JEM-2100F.

Supplementary Figures:

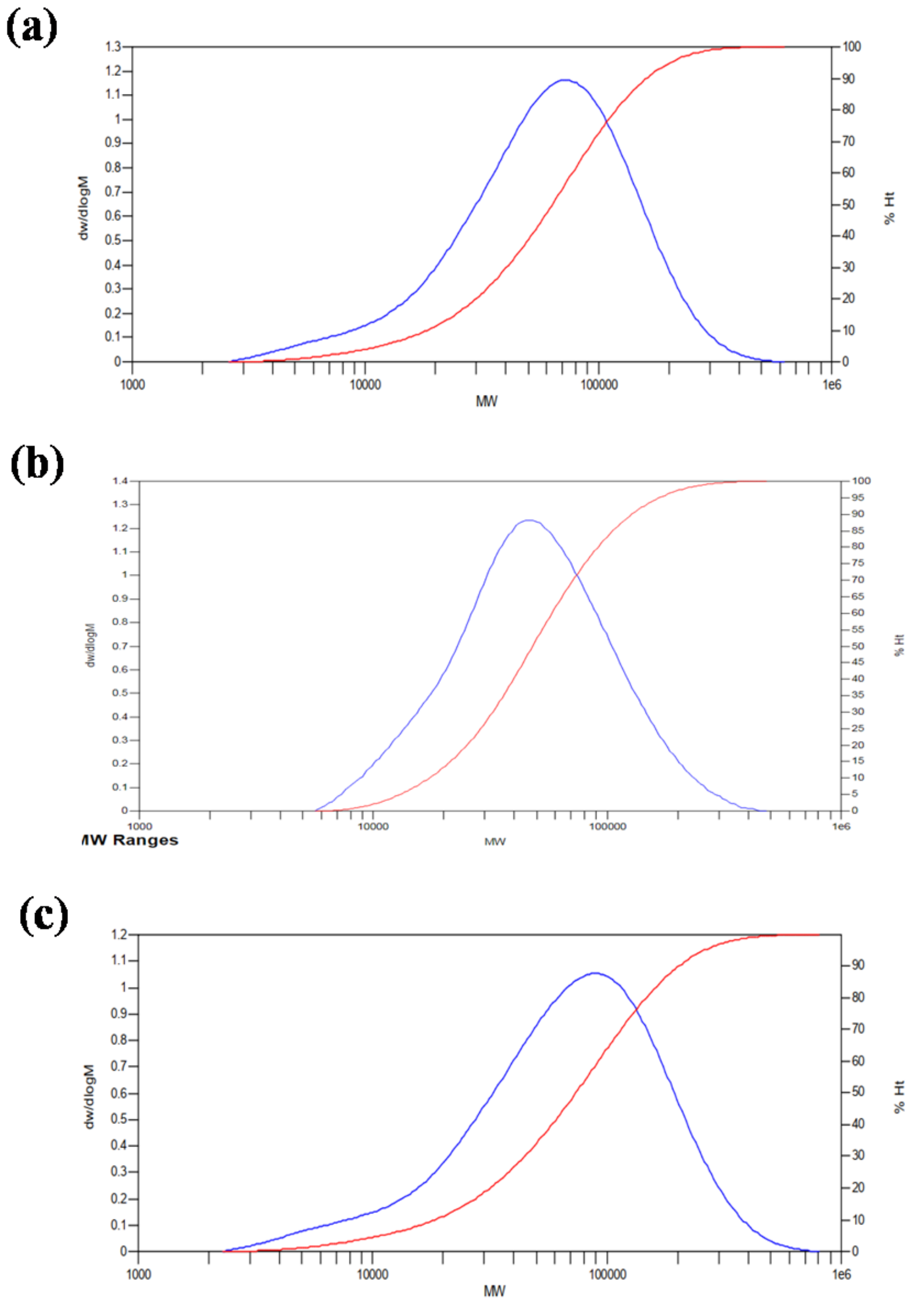

Figure. S1 High temperature gel permeation chromatography (HT-GPC) trace of (a) PM6; (b) PMT-FT-10; (c) PMT-CT-10.

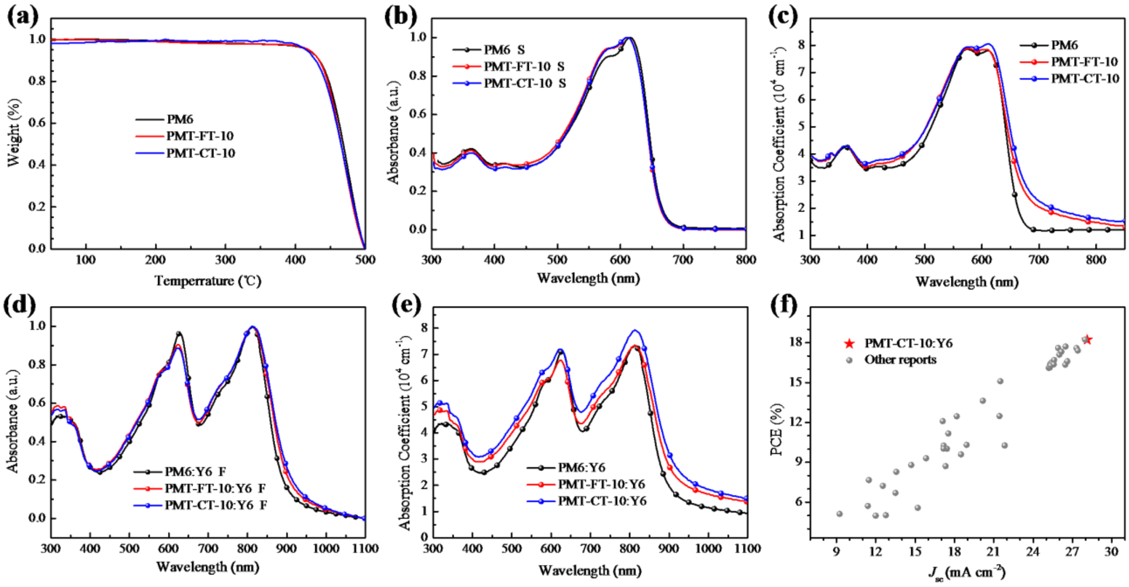

Figure. S2 a) TGA plots of PM6, PMT-FT-10 and PMT-CT-10 with a heating rate of 10℃ min^{-1} under N_2 atmosphere. b) Normalized absorption spectrum of PM6, PMT-FT-10 and PMT-CT-10 in $CHCl_3$ solution (S). c) Absorption coefficients of PM6, PMT-FT-10 and PMT-CT-10 in film state. d) Normalized absorption spectra and e) absorption coefficients of blend films (F) based on polymer donors:Y6. f) Scatter plot of the PCE *vs* J_{sc} for the high-efficient terpolymers-based PSCs reported in literatures and optimized PMT-CT-10:Y6 based device (labeled as stars).

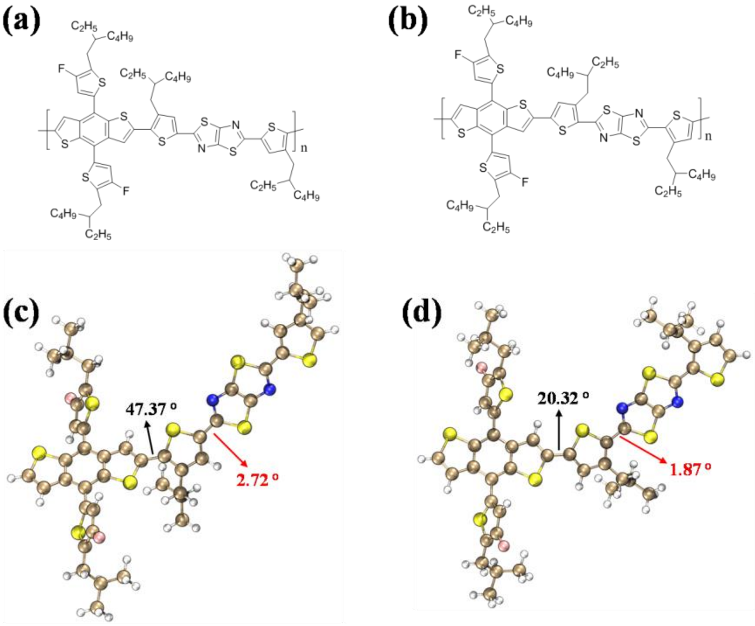

Figure S3. Chemical structures of a) PMT-FT-100, b) PMT-CT-100, and the corresponding torsion angles of the optimal geometries for one unit of c) PMT-FT-10, d) PMT-CT-10, respectively.

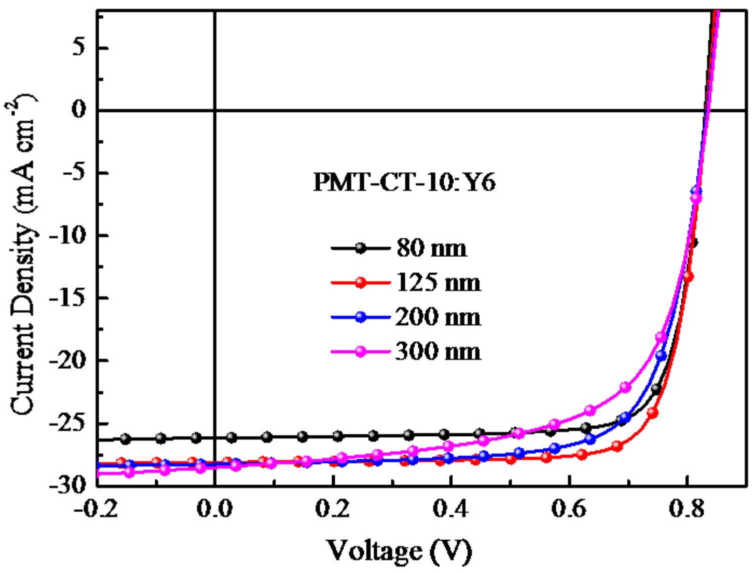

Figure S4. *J−V* curves of the optimized PMT-CT-10-based PSCs with different film thickness under illumination of AM1.5G, 100 mW cm^{-2}.

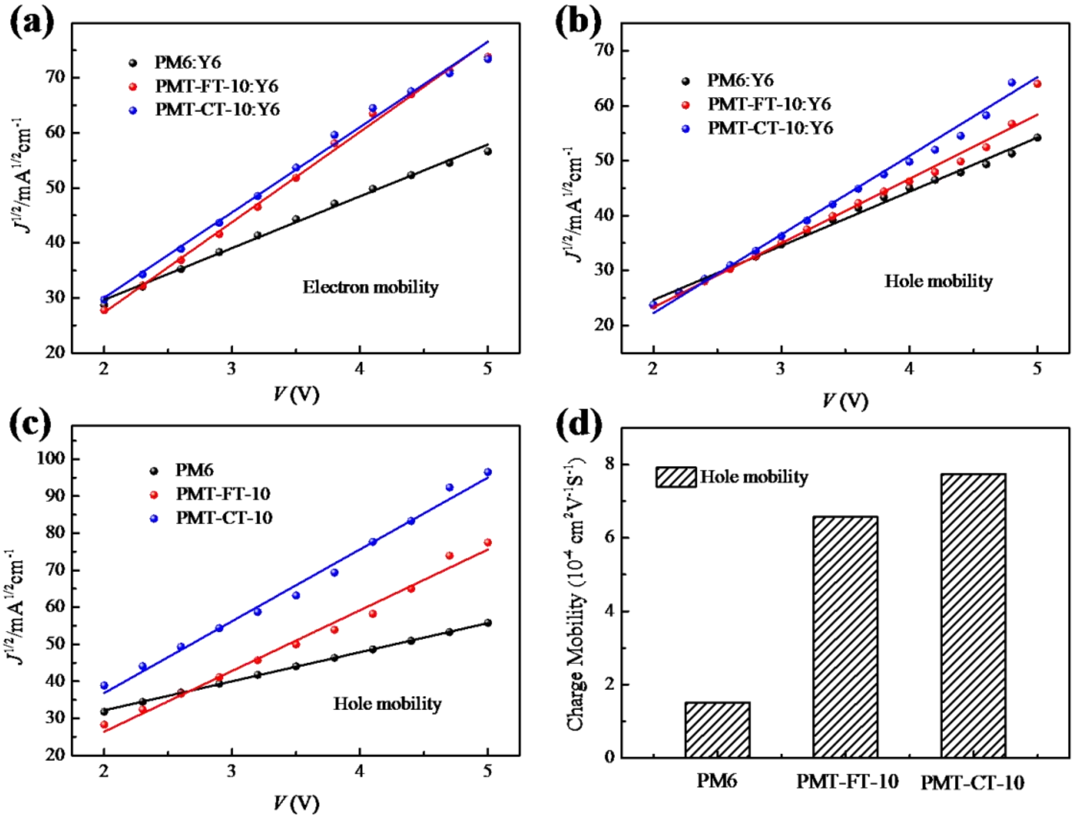

Figure S5. Plots for the measurements of a) electron mobilities and b) hole mobilities of the polymer donors:Y6 blend films. c) hole mobilities of neat polymer films. d) The determined hole mobilities of neat polymer films.

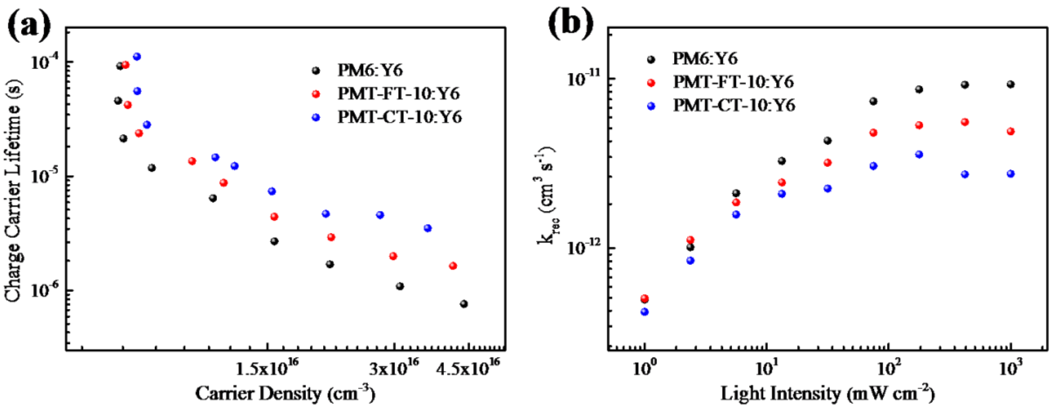

Figure S6. a) Plots of the charge carrier lifetime (τ) *vs* carrier density (n) determined from charge extraction measurements. b) Bimolecular recombination rate constant (k_{rec}) of corresponding PSCs.

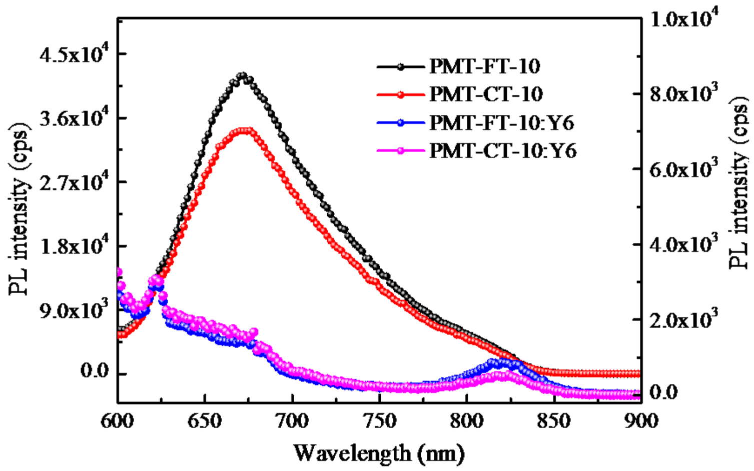

Figure S7. PL spectra of pure polymer donor films and the corresponding blend films of polymer donors:Y6.

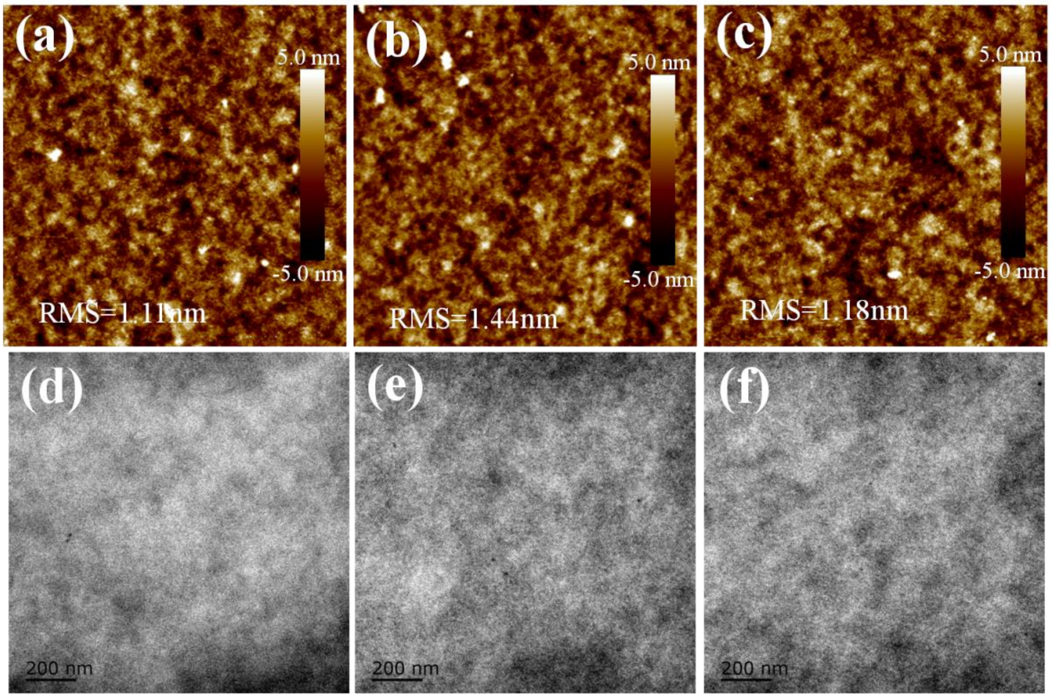

Figure S8. AFM height images (5×5 μm^2) (a-c) and TEM images (scale bar of 200 nm) (d-f) of the blend films based on: a), d) PM6:Y6; b), e) PMT-FT-10:Y6; c), f) PMT-CT-10:Y6.

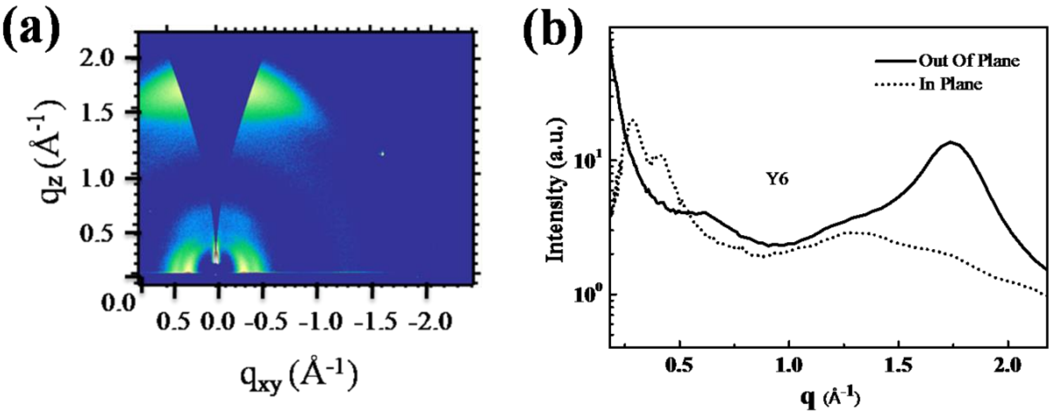

Figure S9. a) 2D-GIWAXS image of the neat films of Y6 and b) corresponding 1D line-cuts in the In-Plane (IP) and Out-Of-Plane (OOP) direction.

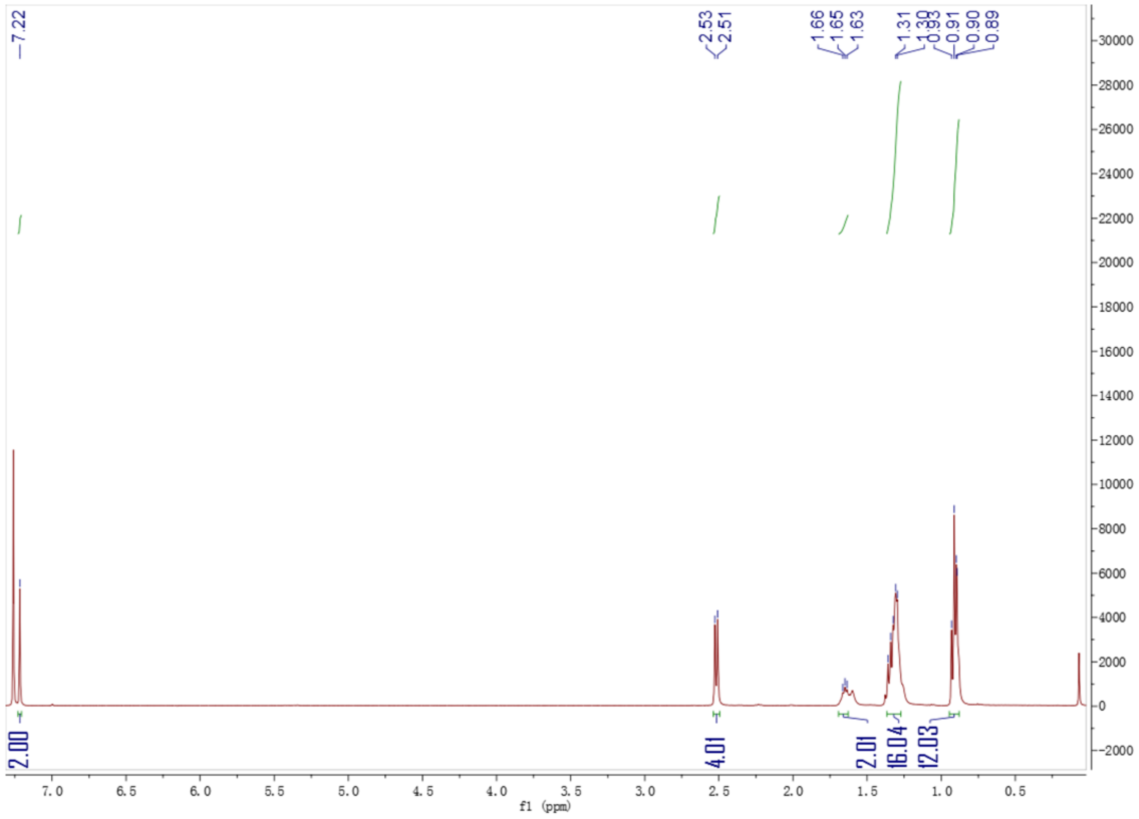

Figure S10. ^{1}H-NMR spectrum of monomer TTz-FT-Br in $CDCl_3$.

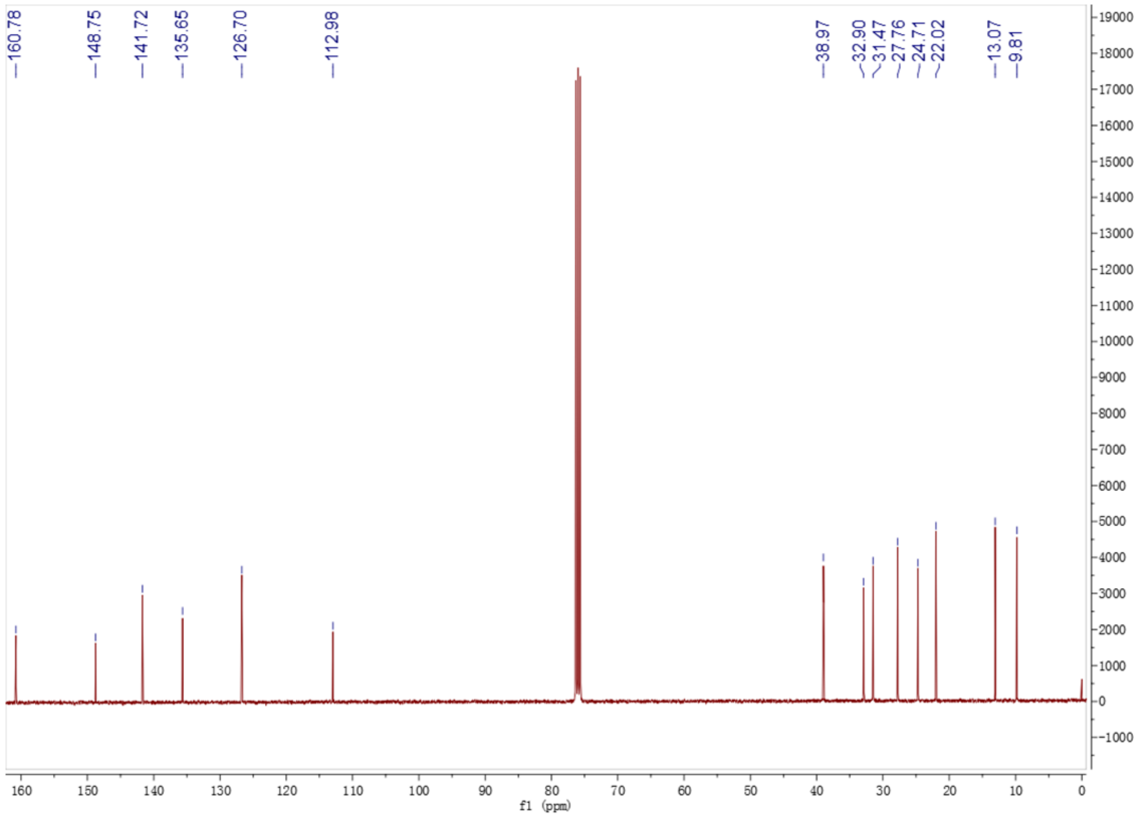

Figure S11. ^{13}C-NMR spectrum of monomer TTz-FT-Br in $CDCl_3$.

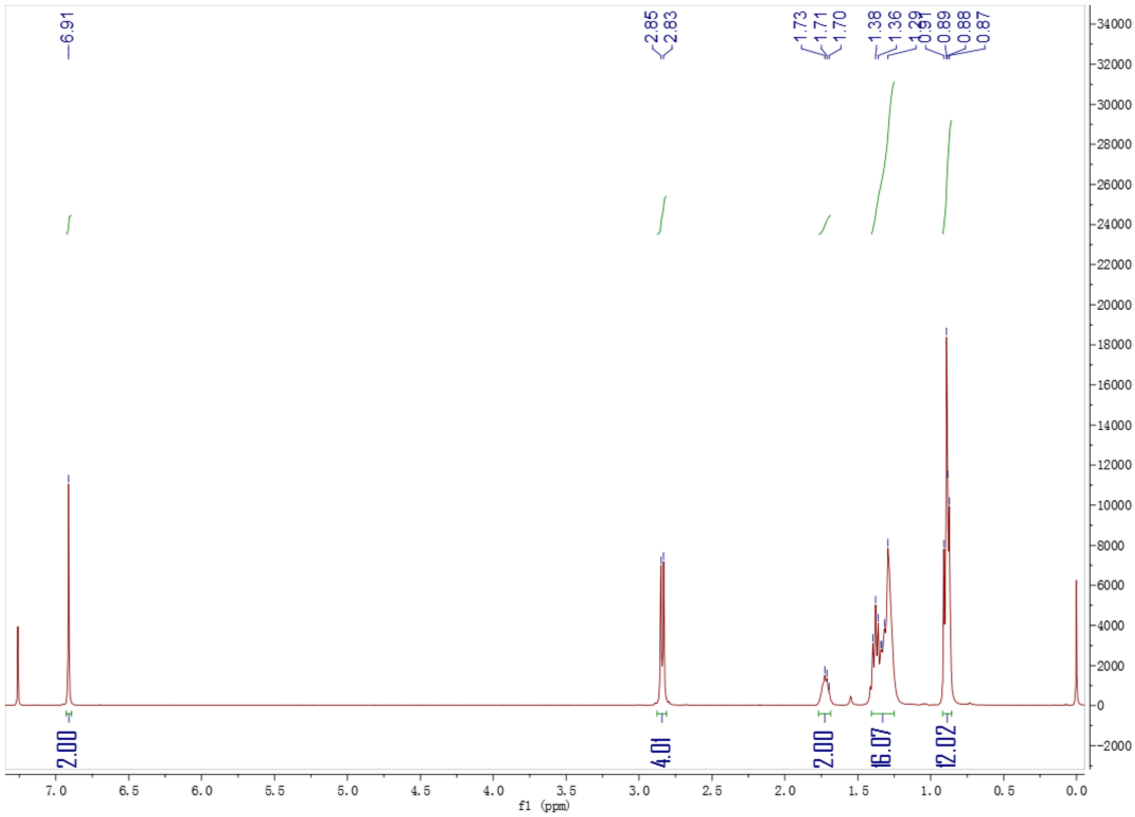

Figure S12. ^{1}H-NMR spectrum of monomer TTz-CT-Br in $CDCl_3$.

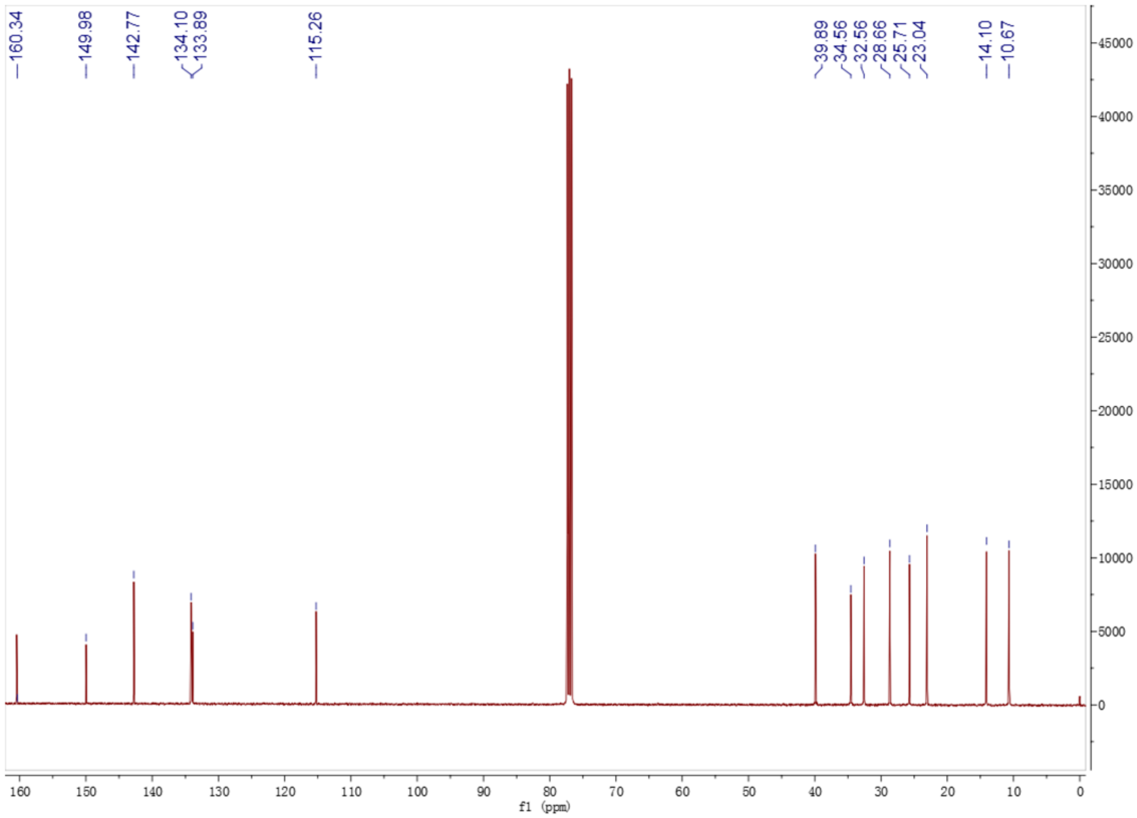

Figure S13. ^{13}C-NMR spectrum of monomer TTz-CT-Br in $CDCl_3$.

Supplementary Tables

Table S1. Photovoltaic parameters of the PSCs based on PMT-CT-10:Y6 blend films with different processing conditions, and the PSCs based on other terpolymer donors with incorporating the TTz-CT units with 20%, 30% and 100% proportions and the TTz-FT units with 20% and 30% proportions.

Donor:acceptor	Weight Ratio	Additive (%)	Annealing temperature[a] (℃)	V_{oc} (V)	J_{sc} (mA cm^{-2})	FF (%)	PCE (%)
PMT-CT-10:Y6	1:1	CN (0.5)	110	0.830	27.51	76.83	17.54
	1:1.2	CN (0.2)	110	0.835	27.35	75.83	17.32
		CN (0.5)	\	0.855	27.16	75.66	17.57
			90	0.842	27.67	75.71	17.64
			110	0.832	28.12	77.85	18.21
			130	0.823	27.84	77.63	17.79
			150	0.808	27.13	74.79	16.70
		CN (0.8)	110	0.827	26.61	78.71	17.32
	1:1.4	CN (0.5)	110	0.833	27.62	77.34	17.79
PMT-CT-20:Y6	1:1.2	CN (0.5)	110	0.837	27.55	77.65	17.91
PMT-CT-30:Y6				0.841	27.34	75.12	17.27
PMT-CT-100:Y6				0.868	22.13	68.75	13.21
PMT-FT-20:Y6	1:1.2	CN (0.5)	110	0.839	26.61	76.33	17.04
PMT-FT-30:Y6				0.845	26.55	74.96	16.82

[a] All devices were fabricated with thermal annealing treatment for 10 min.

Table S2. Summary of the different molecular weight of PMT-CT-10 and corresponding photovoltaic performance of PMT-CT-10-based devices with different batches.

Batches	PMT-CT-10		PMT-CT-10:Y6
	M_n (kDa)	PDI	PCE (%)[a]
Batch 1	22.94	2.88	17.31
Batch 2	31.82	1.87	17.85
Batch 3	37.84	2.49	18.21
Batch 4	43.52	1.89	17.56

[a] All devices were fabricated with optimized conditions.

Table S3. The photovoltaic performances parameters of the optimized PSCs based on PMT-CT-10:Y6 active layer with different film thicknesses.

Active layer	Thickness (nm)	V_{oc}(V)	J_{sc} (mA cm^{-2})	FF(%)	PCE(%)
PMT-CT-10:Y6	80	0.830	26.15	79.20	17.19
	125	0.832	28.12	77.85	18.21
	200	0.833	28.25	72.29	17.01
	300	0.835	28.54	64.86	15.46

Table S4. Charge carrier mobilities of the optimized devices based on polymer donors:Y6 and hole mobilities of the neat polymer donor films.

Blend/Neat films[a]	μ_h (cm^2 V^{-1} s^{-1})	μ_e (cm^2 V^{-1} s^{-1})	μ_e/μ_h
PM6:Y6	2.16×10^{-4}	6.37×10^{-4}	2.95
PMT-FT-10:Y6	6.58×10^{-4}	7.94×10^{-4}	1.21
PMT-CT-10:Y6	8.06×10^{-4}	9.16×10^{-4}	1.14
PM6	1.51×10^{-4}	/	/
PMT-FT-10	6.57×10^{-4}	/	/
PMT-CT-10	7.74×10^{-4}	/	/

[a] The devices based on blend films were fabricated with D/A ratio of 1:1.2 and additive of 0.5% CN.

Table S5. Summary of the GIWAXS parameters for the neat polymer donor films.

Neat polymer donor films	direction	diffraction	q (Å^{-1})[a]	d-spacing (Å)[a]	FWHM (Å^{-1})[a]	CCL (Å)[a]
PM6	Out of plane	010	1.58 (1.58±0.01)	3.98 (3.98±0.02)	0.694 (0.694±0.002)	8.15 (8.15±0.02)
		100	0.28 (0.28±0.01)	22.44 (22.44±0.81)	0.122 (0.122±0.002)	46.49 (46.49±0.76)
	In plane	100	0.28 (0.28±0.01)	22.44 (22.44±0.78)	0.105 (0.105±0.003)	54.06 (54.06±1.55)
PMT-FT-10	Out of plane	010	1.62 (1.62±0.02)	3.88 (3.88±0.05)	0.519 (0.519±0.001)	10.89 (10.89±0.03)
		100	0.28 (0.28±0.01)	22.44 (22.44±0.83)	0.185 (0.185±0.002)	30.65 (30.65±0.33)
	In plane	100	0.29 (0.29±0.02)	21.67 (21.67±1.52)	0.117 (0.117±0.001)	48.53 (48.53±0.42)

PMT-CT-10	Out of plane	010	1.64<br>(1.64±0.03)	3.83<br>(3.83±0.07)	0.503<br>(0.503±0.004)	11.24<br>(11.24±0.09)
		100	0.29<br>(0.29±0.01)	21.67<br>(21.67±0.75)	0.173<br>(0.173±0.003)	32.72<br>(32.72±0.56)
	In plane	100	0.29<br>(0.29±0.02)	21.59<br>(21.59±1.55)	0.104<br>(0.104±0.002)	54.18<br>(54.18±1.05)

[a] The average values with standard deviation were obtained from more than 6 calculations with Gauss.

Table S6. Summary of the GIWAXS parameters for the optimized blend films based polymer donors:Y6.

Blend films	direction	diffraction	q ($Å^{-1}$)[a]	*d*-spacing (Å)[a]	FWHM ($Å^{-1}$)[a]	CCL (Å)[a]
PM6:Y6	Out of plane	010	1.70<br>(1.70±0.01)	3.70<br>(3.70±0.02)	0.321<br>(0.321±0.002)	17.62<br>(17.62±0.11)
	In plane	100	0.29<br>(0.29±0.01)	21.67<br>(21.67±0.73)	0.074<br>(0.074±0.001)	76.42<br>(76.42±1.03)
PMT-FT-10: Y6	Out of plane	010	1.71<br>(1.71±0.02)	3.67<br>(3.67±0.04)	0.318<br>(0.318±0.002)	17.78<br>(17.78±0.12)
	In plane	100	0.30<br>(0.30±0.01)	20.94<br>(20.94±0.71)	0.081<br>(0.081±0.002)	69.81<br>(69.81±1.73)
PMT-CT-10: Y6	Out of plane	010	1.72<br>(1.72±0.03)	3.65<br>(3.65±0.06)	0.310<br>(0.310±0.002)	18.24<br>(18.24±0.12)
	In plane	100	0.31<br>(0.31±0.02)	20.27<br>(20.27±1.33)	0.080<br>(0.080±0.003)	70.69<br>(70.69±2.66)

[a] The average values with standard deviation were obtained from more than 6 calculations with Gauss.
